# Supplementary material for: Solitary and Synergistic Effects of Different Hydrophilic and Hydrophobic Phospholipid Moieties on Rat Behaviors
Source: Pharmaceutics. 2024 Jun 4;16(6):762. doi: 10.3390/pharmaceutics16060762 (PMC11207216; doi:10.3390/pharmaceutics16060762)
Supplement: Supplementary file 1 [file pharmaceutics-16-00762-s001.zip › Table S1.pdf]

Table S1 Particle size of liposomes

Type	n	Particle size (nm)
Cho-lip	3	124.3 ± 4.3
PEG-lip	3	151.6 ± 6.8

Values are mean ± SD.
