# Supplementary figures and images for: Solitary and Synergistic Effects of Different Hydrophilic and Hydrophobic Phospholipid Moieties on Rat Behaviors
Source: Pharmaceutics. 2024 Jun 4;16(6):762. doi: 10.3390/pharmaceutics16060762 (PMC11207216; doi:10.3390/pharmaceutics16060762)

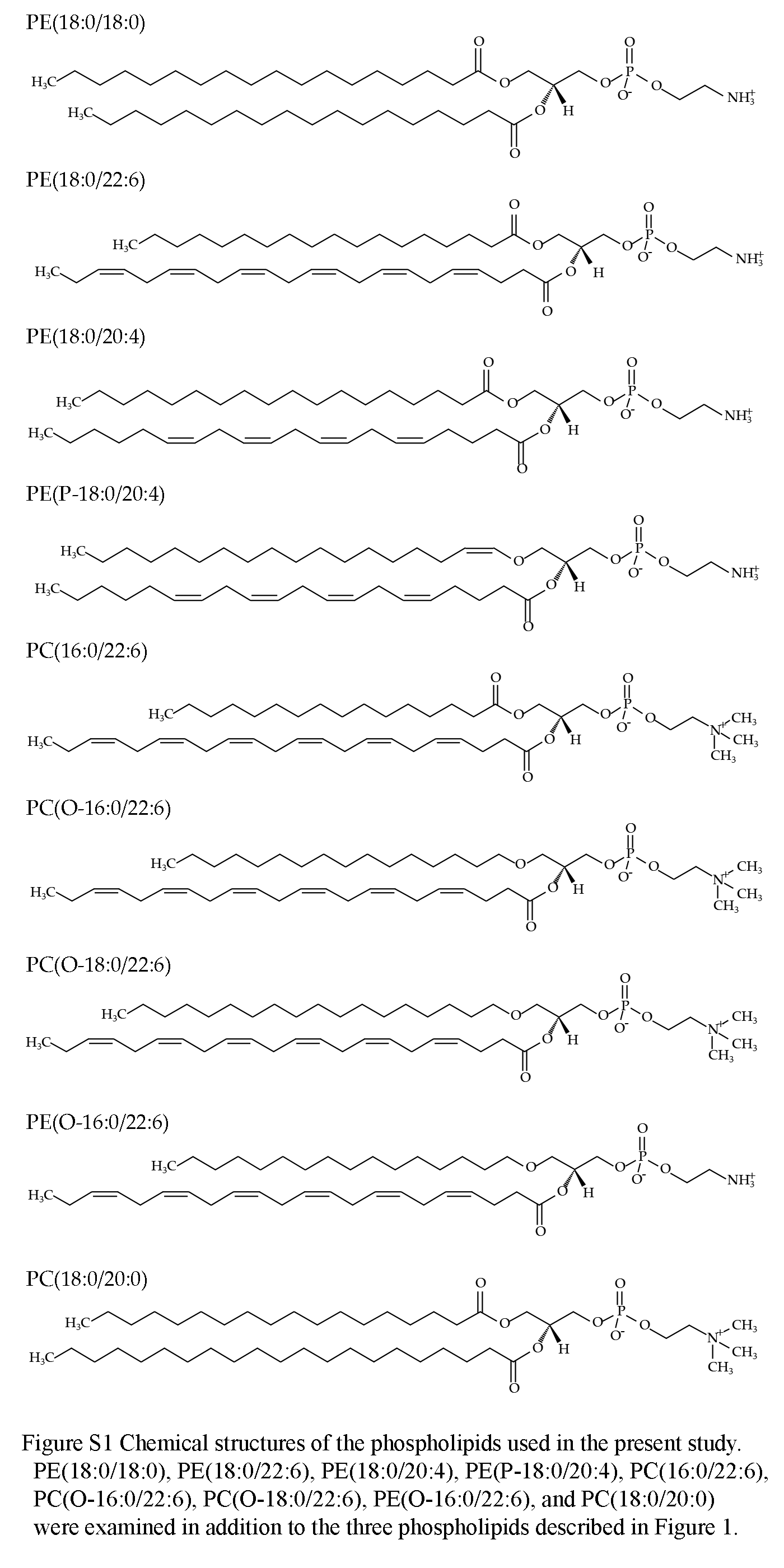

Supplement: Supplementary file 1 [file pharmaceutics-16-00762-s001.zip › Figure S1.tif]

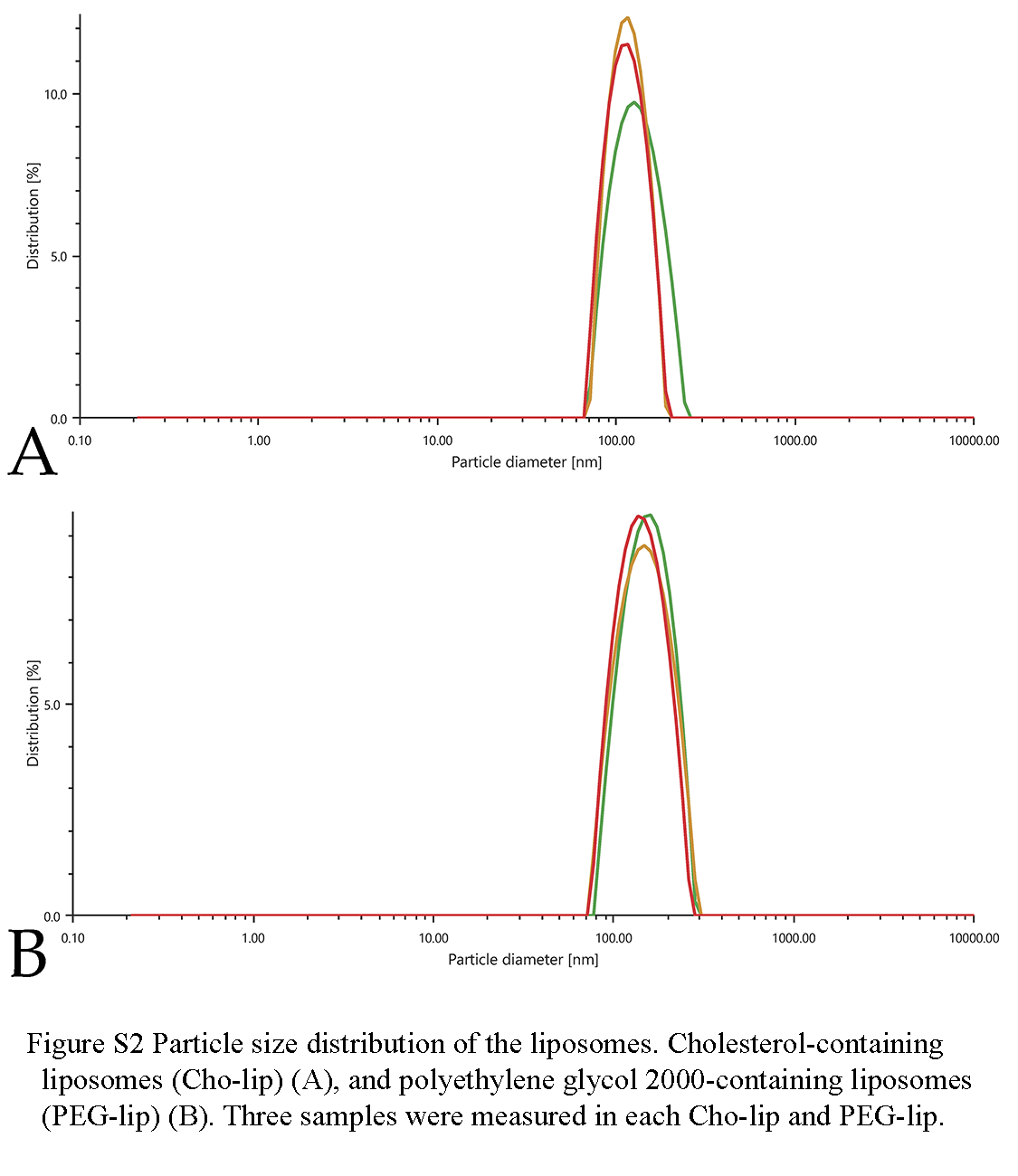

Supplement: Supplementary file 1 [file pharmaceutics-16-00762-s001.zip › Figure S2.tif]
